# Among the Pines at Bournemouth

**Published:** 1889-12-07

**Authors:** 


### Among the Pines at Bournemouth.

Stepping softly upon the warm, dry carpet of needles, we enter the world of pines. There is a hush around—the quiet hush of nature's cathedral. Far up overhead the choir of birds faintly chant their *Te Deum*, and we sing the "amens" under our breath or in our hearts. The silent congregation of countless stately pines surround us close; the odour of their soothing breath comes in healing wafts; their dry warmth wraps round our chilly forms, and we forget for a blissful moment that the time of year—and, perhaps, the time of life also—is winter.

It is unspeakably luxurious, this curious, terebinthine climate, which lulls our pains, be they bodily or mental. Winding in and out among the stiff, straight inhabitants, standing like an army awaiting the word of command to march onwards, we let ourselves go; we release the clenched hold of the reins; we cease jogging that hard-driven steed, our brain, to try and keep up with the reckless pace of to-day. It is as though we have died quietly, easily, and, at once, entered into another and a restful life. How far away the racketting, careering world seems, how pitiful its mad race, how paltry its prizes, after all! Strangely gentle thoughts steal in and about the lodges of our brain. We wonder how we could ever have felt that bitter animosity to some particular fellow-creature who had crossed us; that rankling jealousy of another who stood in front, blocking up our path. Then we ponder, but lazily, on the change, not with storms of repentance, though, over the ill-doings of our old selves. No, we are too dreamily satisfied to be anything but living, breathing, idling, forgetful that our deep contentment is simply born of the odorous whispers and sighs of the dark, silent giants surrounding us.

Presently, there is a little shock. We come, suddenly, face-to-face with a mortal like ourselves, wandering dreamily, whose footsteps tread noiselessly on the thick soft carpet the pines have spread for us. There is a meeting of souls—through their windows, the eyes—just for a brief moment, and we pass on and on through the scented dimness of the wood.

All at once, unexpectedly, we emerge from the pines out on the sandy steeps, and, from them, on to the cliffs hanging over the wide stretch of blue waters dancing in the sunlight. For—doubt it as ye may, ye hapless, fog-bound prisoners of the metropolis in these mirky days of winter—the sun has undisputed possession of the clear skies over this renowned queen of health resorts. Our lungs expand with the pure air of the heights. And we are in—and of—the world again, all our senses on the alert; among our fellows, human interests become keenly alive once more.

There is a long procession—for the most part of invalids—wending their way along the cliff path. Every group has its little history; each bath-chair contains the pivot around which converge the hopes and fears of the handful of anxious attendant ones accompanying it. Here comes one, its occupant a bright young girl, too slim and too spiritual-looking, but all gleeful, because in this balmy, temperate climate she is no longer cooped-up indoors. Like a bird released from its cage, she is giddy with the joy of the open, and her radiant happiness is reflected on the faces of the group surrounding the chair. She is their sun, and, Clytie-like, they live only in her smiles. Truly, the sight is good to see. But they pass, and we glance sadly at a lad with bowed, stooping shoulders and narrow chest. As he raises his face, its sunken, brilliant eyes and pinched features tell out the pathetic fact that his days are numbered. He is the only son of his mother, the widow walking by his side, whose heart is full of thanksgiving that the bracing, though mild, air can so much as lengthen—for even a time—the young life.

Another bath-chair. Its inmate a highly-coloured dowager in sables and velvets; on her lap, a spoilt, petted poodle. Which is the invalid? Behind them a philosophical old donkey draws a soft-eyed mother and her baby. Then, a brace of fashionable girls swing along with bright faces, flourishing with manly air their walking-sticks. Next, a lone old man with no friend but his wiry terrier creeps slowly, and so on.

Turning our eyes on the drive that runs parallel with the cliff path, we see more life-phases. There, among the line of carriages rolling along, is a handsome equipage with resplendent liveries and fine horses. In strange, dreary mockery the owner lies stretched out, flat and helpless, on a narrow mattress, a rich man brought to the land of pines that his span may be lengthened out to its utmost limits. As he lies there, his fading eyes fixed upon the far-away Needles—suddenly lit up by a sidelong glint of the afternoon sun, and standing out from the main, one, two, three!—what are his thoughts? Perhaps of the cruel worthlessness of his gold, its utter inability to buy for him a longer lease of life.

One and all, the passing and repassing crowds are seekers after health, finding it in the wondrously invigorating breezes with their curative properties, the pine odours, and the peculiarly dry atmosphere, all of which tend unmistakeably to build up their dilapidated frames, soothe their nervous systems, lull their rheumatic and gouty pangs—in short, either to turn them out hale and sound or, at least, to stave off the dreaded end.

Happy for them that in their own island home they can find such a resort, instead of being driven into the far-off exile—of rarest loveliness, but, notwithstanding, exile still—of the Riviera, where the heart-hunger for home dims the surrounding radiance of sea, and sky, and land.

